# Supplementary material for: Diversity, taxonomic composition, and functional aspects of fungal communities in living, senesced, and fallen leaves at five sites across North America
Source: PeerJ. 2016 Dec 13;4:e2768. doi: 10.7717/peerj.2768 (PMC5157190; doi:10.7717/peerj.2768)

**Supplemental Figures**

**Supplemental Figure 1.** Species accumulation curves (Mao Tau; solid line), 95% confidence intervals (short dash), and bootstrap estimates of richness (long dash) based on ITS-partial LSU rDNA for endophytic and saprotrophic fungi from each of five N. American sites (AZC: Chiricahua Mountains, Arizona; NCH: Highlands Biological Station, North Carolina; FLA: Archbold Biological Station, Florida; AKE: Eagle Summit, Alaska; AKN: Nome, Alaska).

**
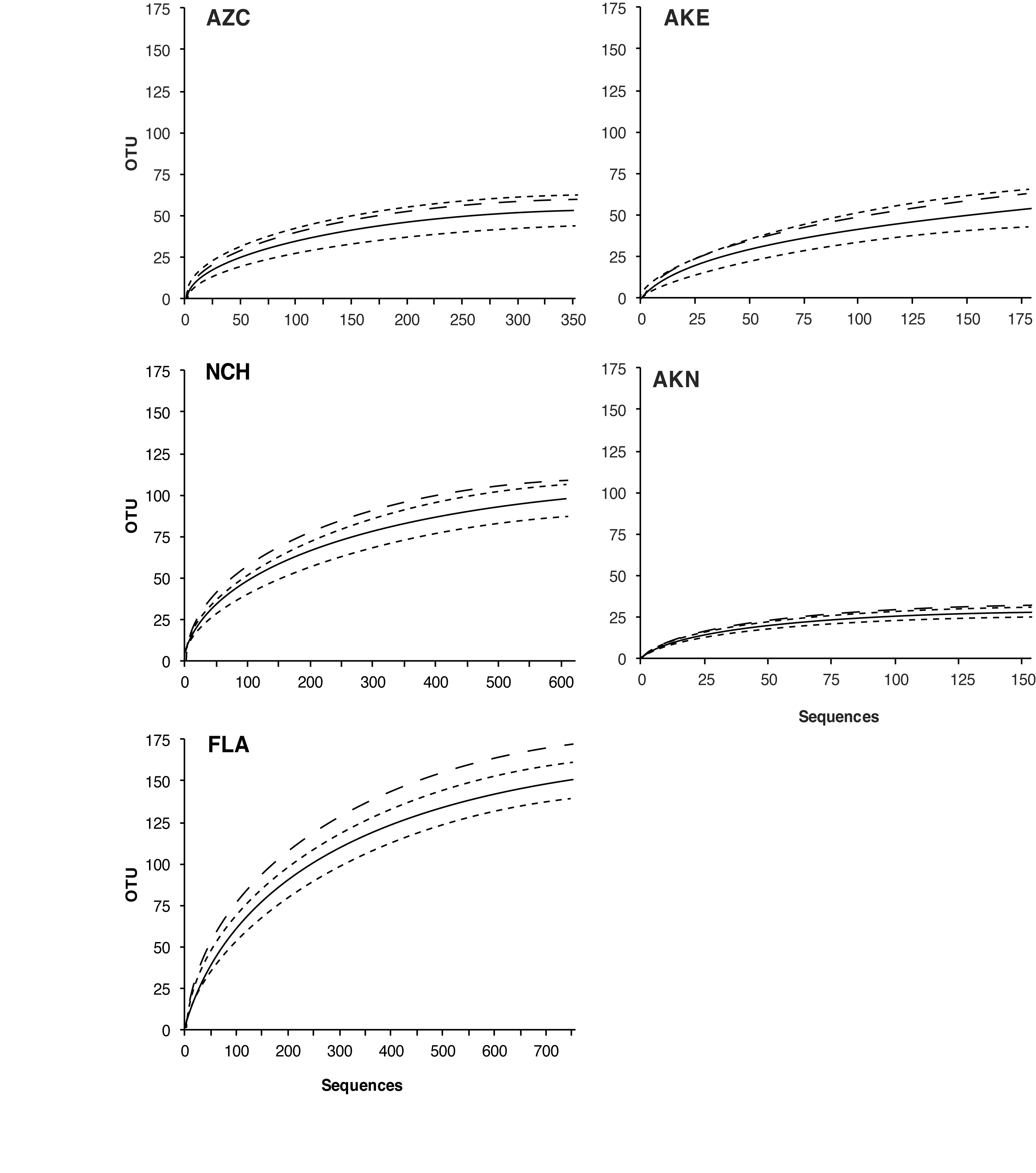
**

**Supplemental Figure 2.** Non-metric multidimensional scaling (NMDS) analysis of culturable fungal communities as a function of host and leaf type in (A) five sites across North America; (B) AZC; (C) NCH; and (D) FLA. Each point represents the fungal community from a single plant/leaf-type combination. Convex hulls (i.e., the smallest convex polygon containing all points) show the area occupied by points from each host or site. One-way ANOSIM indicates community composition differs significantly across sites (A) and host species (B-D). However, post-hoc pairwise comparisons indicate no significant differences in communities from *Pinus elliottii* and *P. clausa* in FLA, *Serenoa repens* and *Quercus inopina* in FLA, and *Quercus rugosa* and *P. arizonica* in AZC (P>0.05). The Morisita-Horn index was used to quantify community similarity for NMMDS and ANOSIM. Singleton OTU and hosts from which <4 isolates were sequenced were excluded from analyses. Site-specific panels are not given for AKN and AKE because samples sizes were too low per host individual.


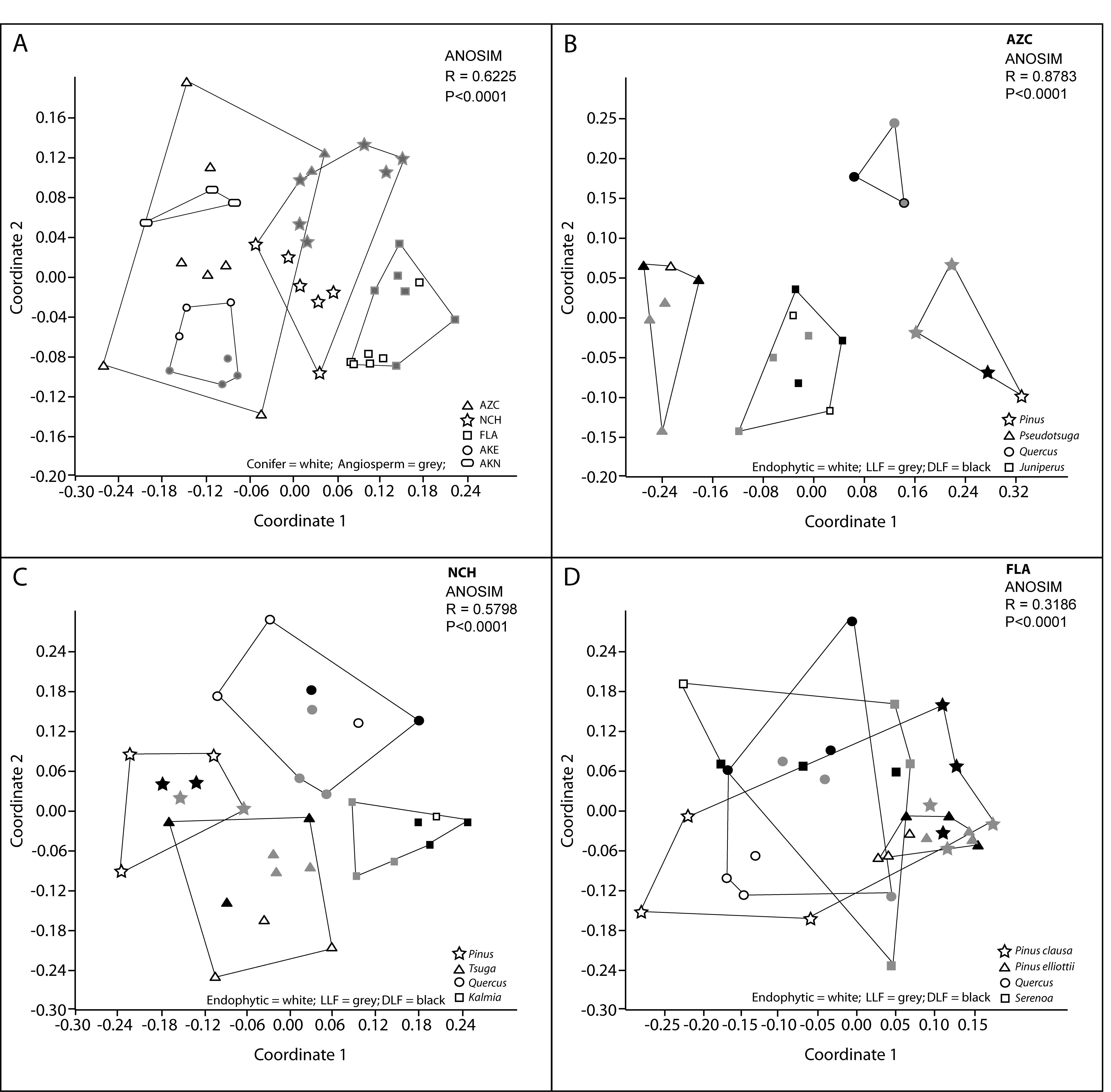


**Supplemental Figure 3.** Venn diagram illustrating the overlap of non-singleton OTU (based on 95% ITS-partial LSU rDNA sequence similarity) among living leaves (Endo), dead leaves in the canopy (DLF), and leaf litter (LLF), followed by the percentage of OTU (out of 236 total non-singleton OTU) in parentheses. The number of singleton OTU from each leaf type are shown in square brackets.


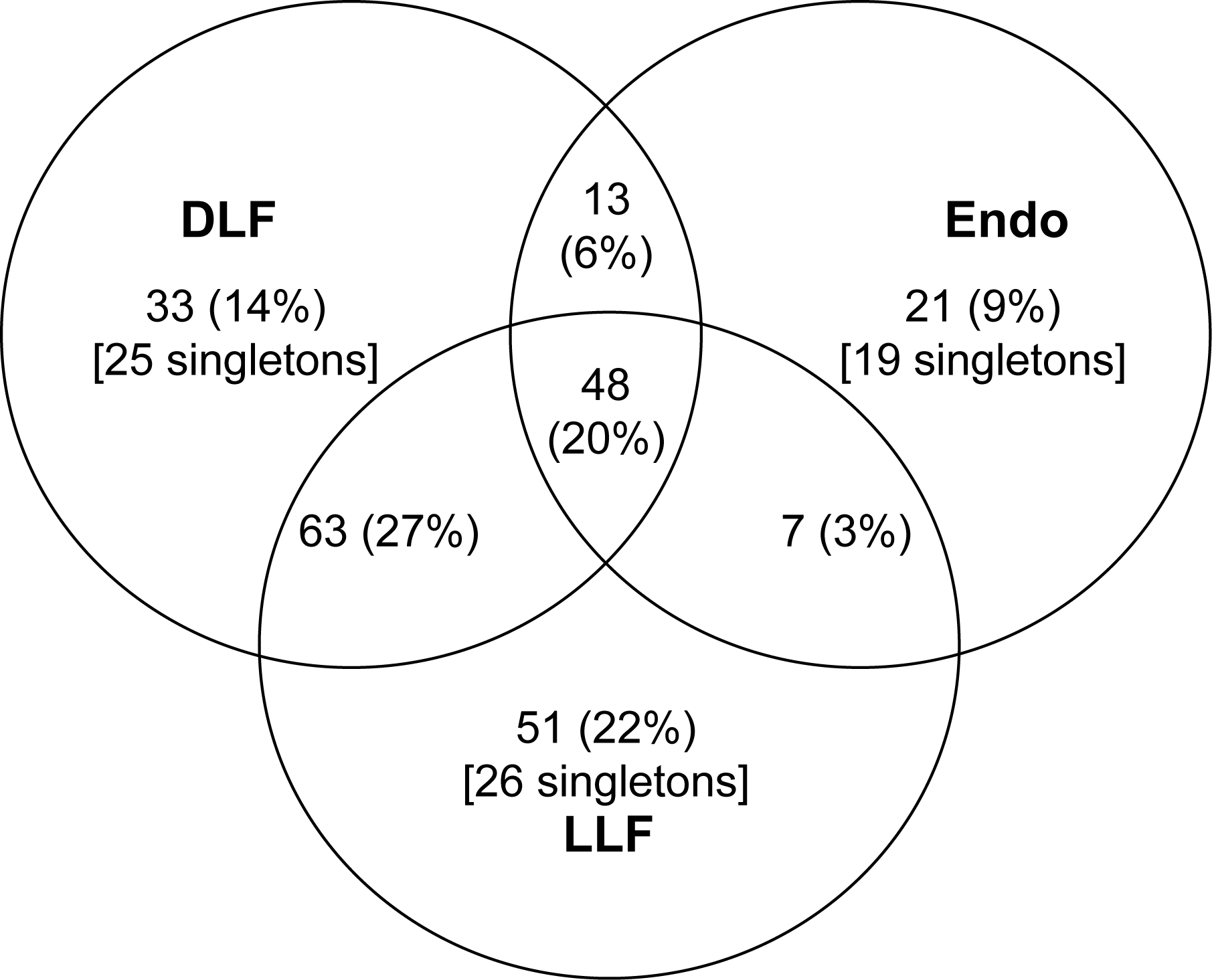

Supplement: Supplemental Information 1 [file peerj-04-2768-s001.doc]
