# Supplementary material for: Diversity, taxonomic composition, and functional aspects of fungal communities in living, senesced, and fallen leaves at five sites across North America
Source: PeerJ. 2016 Dec 13;4:e2768. doi: 10.7717/peerj.2768 (PMC5157190; doi:10.7717/peerj.2768)
Supplement: Table S1 — Similarity (1- variably weighted Odum dissimilarity, DwOdum) between fungal communities in different leaf types for five N. American sites as a function of different alpha weighting factors. At α = 1, DwOdum is equal to DOdum; when α < 1, the influence of abundant taxa will be down-weighted; and when α > 1, the influence of low abundance taxa will be down-weighted. Low sample size of endophytes in AKE precluded comparisons of endophyte and DLF/LLF. [file peerj-04-2768-s006.docx]

| **Supplemental Table 1. Similarity (1- variably weighted Odum dissimilarity, D_wOdum_) between fungal communities in different leaf types for five N. American sites as a function of different alpha weighting factors. At α = 1, D_wOdum_ is equal to D_Odum_; when α <1, the influence of abundant taxa will be down-weighted; and when α >1, the influence of low abundance taxa will be down-weighted. Low sample size of endophytes in AKE precluded comparisons of endophyte and DLF/LLF.** | | | | | | | | | | | | | | |
| --- | --- | --- | --- | --- | --- | --- | --- | --- | --- | --- | --- | --- | --- | --- |
|  |  | **alpha (0)** | | **alpha (1)** | | | **alpha (2)** | | | **alpha (3)** | | **alpha (4)** | | |
|  |  | **1-D_wOdum_** | **p-value** | | **1-D_wOdum_** | **p-value** | | **1-D_wOdum_** | **p-value** | **1-D_wOdum_** | **p-value** | | **1-D_wOdum_** | **p-value** |
| **Endo vs DLF** | **AZC** | **0.13** | <0.001 | | **0.36** | <0.001 | | **0.45** | <0.001 | **0.47** | <0.001 | | **0.48** | <0.001 |
|  | **NCH** | **0.09** | <0.001 | | **0.27** | <0.001 | | **0.43** | <0.001 | **0.53** | <0.001 | | **0.59** | <0.001 |
|  | **FLA** | **0.15** | <0.001 | | **0.30** | <0.001 | | **0.36** | <0.001 | **0.35** | <0.001 | | **0.32** | <0.001 |
|  | **AKE** | NA | NA | | NA | NA | | NA | NA | NA | NA | | NA | NA |
|  | **AKN** | **0.13** | <0.001 | | **0.27** | <0.001 | | **0.23** | <0.001 | **0.15** | <0.001 | | **0.10** | <0.001 |
|  |  |  |  | |  |  | |  |  |  |  | |  |  |
| **Endo vs. LLF** | **AZC** | **0.16** | <0.001 | | **0.30** | <0.001 | | **0.26** | <0.001 | **0.16** | <0.001 | | **0.09** | <0.001 |
|  | **NCH** | **0.08** | <0.001 | | **0.18** | <0.001 | | **0.22** | <0.001 | **0.23** | <0.001 | | **0.24** | <0.001 |
|  | **FLA** | **0.12** | <0.001 | | **0.25** | <0.001 | | **0.24** | <0.001 | **0.18** | <0.001 | | **0.15** | <0.001 |
|  | **AKE** | NA | NA | | NA | NA | | NA | NA | NA | NA | | NA | NA |
|  | **AKN** | **0.16** | <0.001 | | **0.26** | <0.001 | | **0.14** | <0.001 | **0.05** | <0.001 | | **0.02** | <0.001 |
|  |  |  |  | |  |  | |  |  |  |  | |  |  |
| **DLF vs. LLF** | **AZC** | **0.24** | <0.001 | | **0.40** | <0.001 | | **0.45** | <0.001 | **0.47** | <0.001 | | **0.49** | <0.001 |
|  | **NCH** | **0.19** | <0.001 | | **0.28** | <0.001 | | **0.37** | <0.001 | **0.46** | <0.001 | | **0.51** | <0.001 |
|  | **FLA** | **0.28** | 0.003 | | **0.53** | <0.001 | | **0.67** | <0.001 | **0.68** | <0.001 | | **0.68** | <0.001 |
|  | **AKE** | **0.22** | 0.024 | | **0.41** | 0.003 | | 0.62 | 0.097 | 0.76 | 0.401 | | 0.83 | 0.494 |
|  | **AKN** | **0.21** | 0.034 | | 0.46 | 0.116 | | 0.60 | 0.189 | 0.66 | 0.251 | | 0.68 | 0.286 |
