# Supplementary material for: Diversity, taxonomic composition, and functional aspects of fungal communities in living, senesced, and fallen leaves at five sites across North America
Source: PeerJ. 2016 Dec 13;4:e2768. doi: 10.7717/peerj.2768 (PMC5157190; doi:10.7717/peerj.2768)
Supplement: Table S3 — Summary of statistical analyses for OTU abundance and functional assays [file peerj-04-2768-s008.docx]

| **Supplemental Table 3. Summary of statistical analyses for OTU abundance and functional assays** | |  |
| --- | --- | --- |
| **Statistical test** | **Results** | |
| **Linear regression comparing abundance of OTU from leaf litter vs. abundance of the same OTU in living leaves** | log transformation: R^2^ = 0.03, F_1,43_ = 1.41, P = 0.2415; lack-of-fit F-test F_12,31_ = 2.43, P = 0.0234 | |
| **Mantel test for correlation between differences in fungal growth on carbon sources vs. ITS-partial LSU rDNA genetic distances** | cellulose: r = 0.060, P = 0.166; lignin: r = 0.096, P = 0.097; pectin: r = 0.122, P = 0.032 | |
| **Test for differences in detectable cellulolytic activity vs. no cellulolytic activity as a function of leaf type of origin** | Fisher’s exact test for endophytes, DLF, and LLF; P = 0.5765 | |
| **Non-parametric test for differences in cellulolytic activity as a function of fungal class (Sordariomycetes, Leotiomycetes, Dothideomyctes, Pezizomycetes)** | Wilcoxon rank sum test: χ^2^_3_= 4.67 P = 0.0071 | |
| **Non-parametric test for differences in cellulolytic activity as a function of leaf type of origin** | Sordariomycetes, Wilcoxon rank sum: χ2_1_= 0.0014 P = 0.9704  Dothideomycetes, Wilcoxon rank sum: χ^2^= 5.72 P = 0.0168 | |
| **Non-parametric test for differences in growth on different carbon sources activity as a function of leaf type of origin** | Sordariomycetes, Wilcoxon rank sum: cellulose: χ^2^ = 0.046, P = 0.8311; lignin: χ^2^ = 2.91, P = 0.0879; pectin: χ^2^ = 1.47, P = 0.2258  Dothideomycetes, Wilcoxon rank sum: cellulose: χ^2^ = 7.38, P = 0.0066; lignin: χ^2^ = 9.00, P = 0.0027; pectin: χ^2^ = 11.39, P = 0.0007 | |
| **Mantel test for correlation between differences in cellulolytic activity** **vs. ITS-partial LSU rDNA genetic distances** | r = -0.015, P = 0.178 | |
